# Long-Term Outcomes of Autologous Hematopoietic Stem Cell Transplant (HSCT) for Multiple Myeloma: While New Horizons Emerge, It Is Still Only a Silver Lining for Resource-Constrained Settings

**DOI:** 10.7759/cureus.36642

**Published:** 2023-03-24

**Authors:** Saquib Z Banday, Faisal Guru, Maniza Ayub, Syed N Ahmed, Aaqib Z Banday, Mohmad H Mir, Rahila Nisar, Saleem Hussain, Gull M Bhat, Sheikh A Aziz

**Affiliations:** 1 Department of Medical Oncology, State Cancer Institute, Sher-i-Kashmir Institute of Medical Sciences, Srinagar, IND; 2 Department of Medical Oncology, Pediatrics Unit, State Cancer Institute, Sher-i-Kashmir Institute of Medical Sciences, Srinagar, IND; 3 Department of Pathology, Sher-i-Kashmir Institute of Medical Sciences, Srinagar, IND; 4 Department of Pediatrics, Government Medical College, Srinagar, IND; 5 Department of Microbiology, Sher-i-Kashmir Institute of Medical Sciences, Srinagar, IND; 6 Department of Laboratory Hematology, Sher-i-Kashmir Institute of Medical Sciences, Srinagar, IND

**Keywords:** review, transplant, stem cell, myeloma, hematopoietic, bone marrow transplant, autologous transplant

## Abstract

Background

Significant hurdles impede the optimal implementation of hematopoietic stem cell transplantation (HSCT) in low-middle income countries (LMICs). Herein, we highlight the challenges faced in LMICs while performing HSCT and report the long-term outcomes of patients with newly diagnosed multiple myeloma (MM) who underwent autologous HSCT (AHSCT) at our center. Besides, we provide a comprehensive review of studies reporting long-term outcomes of AHSCT in MM from the Indian subcontinent.

Methodology

This study was conducted at the State Cancer Institute, Sher-i-Kashmir Institute of Medical Sciences, Srinagar, India. Case records of all patients with MM who received AHSCT from December 2010 to July 2018 were reviewed retrospectively. A non-systematic literature search was performed using PubMed and Google Scholar databases. Data regarding clinicopathological parameters and long-term follow-up were extracted from relevant studies and for patients included in our study.

Results

At our center, 47 patients (median age 52.0 years) with MM underwent AHSCT. Majority of patients had stage III disease (ISS) and median time to transplant was 11.5 months. The five-year progression free survival (PFS) and overall survival (OS) were 59.1% and 81.2%, respectively. Studies from the Indian subcontinent have observed a five-year OS of ~50% to ~85%. However, a greater variability in the five-year PFS has been reported, ranging from ~20% to ~75%. The median time to transplant has ranged from seven to 17 months (indicating time delays) with median CD34 cell counts of 2.7-6.3×10^6^ cells/kg (lower than developed countries).

Conclusions

Despite significant resource limitations in LMICs, AHSCT is increasingly been performed in MM with encouraging long-term outcomes.

## Introduction

Blood and marrow stem cell transplantation (BMSCT) has seen a revolution, especially in the last decade, with an ever-increasing number of patients being transplanted the world over [1]. Not only has the pool of matched related/unrelated donors expanded but alternative sources of graft including haploidentical donors, unmatched related donors, and umbilical cord blood hematopoietic stem cells are also being increasingly utilized. Also, significant pharmacological advances, improvements in laboratory techniques, expansion of health care services, and a better understanding of genetic/immunological processes (associated with hematopoietic stem cell transplantation [HSCT]) have resulted in a superior and more optimal application of BMSCT in routine clinical practice [2]. Despite these advancements, data from Worldwide Network for Blood and Marrow Transplantation has highlighted the significant differences in transplant rates across the globe (lowest in resource-limited settings) [1]. Besides, the density of HSCT performing centers/teams is significantly lesser in developing nations compared to the developed world [3]. As highlighted in this study, even undertaking an autologous BMSCT (as for multiple myeloma [MM]) remains a big challenge in such settings (such as Africa and parts of Asia).

MM, a clonal plasma cell dyscrasia, is the third most common hematological malignancy [4] accounting for ~10% of all hematological cases [5]. In 2020, ~0.18 million patients were newly diagnosed to have MM [6]. In transplant-eligible patients with MM, autologous HSCT (AHSCT) is now an established treatment modality across all risk subtypes. AHSCT results in a deeper response including minimal residual disease (MRD) negativity, better progression-free survival (PFS), and improves overall survival (OS) in high-risk cases [7]. In addition to routine AHSCT, salvage autologous/allogenic transplant in relapsed settings have been incorporated into the transplant programs for MM in developed countries [8]. Besides, analysis of cancer cell cytogenetics, utilization of CXCR4 inhibitors for mobilization of stem cells, and cryopreservation of the stem cells are routinely undertaken in these centers. As shown by the Center for International Blood and Marrow Transplant Research data, age cutoffs or impaired renal function are no longer absolute contraindications for AHSCT in MM [9,10]. Additionally, performing an AHSCT on an outpatient basis is emerging as a safe, feasible, and cost-effective option with the potential of having better clinical outcomes than an inpatient AHSCT [11]. Unfortunately, significant hurdles impede the optimal implementation of AHSCT for MM in low-middle-income countries (LMICs, defined according to the World Bank classification) including India (most of the previously mentioned measures [e.g. cryopreservation, tumor cytogenetics, etc.] are sparingly employed) [12]. In the year 2018, ~9000 and ~15,000 AHSCTs were performed for MM in the US and Europe, respectively [13,14]. Contrastingly, the total number of AHSCTs performed for MM in India for the years 1983-2016 was less than 3000 [15]. Nonetheless, one of the largest registries for transplant data in India has shown promising trends over the past 10 years with AHSCT numbers for MM increasing from 134/year to 734/year [15]. Similar improving trends have also been noted in other developing countries [12]. Encouragingly, data also suggests AHSCT for MM to be cost-effective and beneficial in outcome despite the resource constraints faced in LMICs [16,17]. In this study, we report the long-term outcomes of patients with MM who underwent AHSCT at our center. Besides, we provide a comprehensive review of studies reporting long-term outcomes of AHSCT in MM from the Indian subcontinent. We highlight the challenges faced in resource-constraint settings while performing a BMSCT and ensuring its best possible long-term outcomes. While new horizons of diagnostic, therapeutic, and prognostic advancements emerge in developed countries, it is only a silver lining in resource-poor countries.

## Materials and methods

This was an observational study conducted at the Department of Medical Oncology, State Cancer Institute (SCI), Sher-i-Kashmir Institute of Medical Sciences, Srinagar, India. The Institutional Ethics Committee of Sher-i-Kashmir Institute of Medical Sciences issued approval 131/IEC-SKIMS/2021. SCI is the only dedicated tertiary cancer care institution in the northernmost part of India (with geographically challenging Himalayan terrain). More than 5000 newly diagnosed patients with cancer are registered and treated annually at this center. This is the only hospital that performs HSCT in this region. All patients with MM who received AHSCT from December 2010 to July 2018 were included. Case records of all these patients were reviewed retrospectively. Data regarding clinicopathological characteristics, transplantation parameters, and follow-up events were extracted. None of our patients with MM received tandem or allogenic HSCT (including cord blood HSCT). Besides, we did not perform AHSCT in any patient with disease relapse/progression (previously treated with or without HSCT). 

AHSCT protocol

Standard evaluation post-induction therapy was done in all patients. All patients were assessed as per Charlson Comorbidity Index (age-adjusted) for medical eligibility (cutoff <6) before proceeding ahead with AHSCT. Stem cells were harvested from peripheral blood after mobilization with granulocyte colony-stimulating factor (G-CSF) with or without plerixafor. Standard doses G-CSF (10 μg/kg/day in two divided doses for four days [days -3 to -6]) and plerixafor (0.24 mg/kg/day, for one or two days, eight to 10 hours before apheresis [day -2 or -1]) were used for mobilizing the stem cells [18]. Depending on the CD34 cell count, one or two collections using the COBE Spectra apheresis machine (Terumo, Tokyo, Japan) were done (days -1 ± -2, respectively). CD34 cell count was measured using flow cytometry and values of >2.0×10^6^ cells/kg were considered optimal [19]. Collected stem cells were stored (maximum 36-48 hours) at 4°C (cryopreservation was not used) and viability testing of the stem cells was not performed before infusion [20]. Myeloablative conditioning using melphalan at 140-200 mg per m^2^ (with appropriate renal modification) as an intravenous infusion over 30 minutes (on day -1) was used after stem cell mobilization [21].

Harvested stem cells were infused intravenously approximately 24 hours after administration of melphalan with routine infusion sets (labeled day 0) [22]. Post stem cell infusion, all patients were continued with G-CSF (at a dose of 5 μg/kg/day) from day +1 until the physician's discretion [23]. All patients were started on antifungal prophylaxis with posaconazole [24] and antiviral prophylaxis with low-dose acyclovir [25]. No prophylactic antibacterial therapy was used. Following AHSCT, all patients received standard maintenance therapy according to the institutional protocol.

Neutrophil engraftment was defined as the attainment of persistent absolute neutrophil counts of >0.50×10^9^/L [26]. Platelet counts of >20×10^9^/L for at least a week (in absence of platelet transfusion) signified platelet engraftment [26,27]. For our study, time to transplant was defined as the interval (in months) from the date of diagnosis to day 0 of AHSCT. Transplant-related mortality (TRM) was defined as a fatal outcome occurring within day +100 of AHSCT.

Statistical analysis

All data extracted was entered into a predesigned datasheet. Shapiro-Wilk test and inspection of normality plots were utilized for the assessment of data distribution. Quantitative data have been expressed as mean±standard deviation (parametric) or median (25th, 75th percentile) (non-parametric). Qualitative data are provided as percentages (n=a) where ‘a’ signifies the number of patients with the said characteristic. Time interval (in years) from AHSCT (day 0) to death or last follow-up represented OS which has been expressed as five-year survival with 95% confidence intervals (95% CI). PFS was the time in years from AHSCT (day 0) to disease relapse, death, or last follow-up (expressed as five-year PFS with 95% CI). The Log Rank test was used to compare survival differences between variables (threshold for statistical significance: p=<0.05). Statistical Package for Social Sciences software (SPSS, version 23; IBM Corp., Armonk, NY, USA) was utilized for performing statistical analysis.

Search strategy

The PubMed/Medline and Google Scholar databases were used to identify studies from the Indian subcontinent reporting treatment of MM with AHSCT (published till September 2022). Keywords utilized for our non-systematic literature search were ‘myeloma’, ‘multiple myeloma’, ‘autologous transplant’, ‘HSCT’, ‘stem cell transplant’, ‘India’, ‘Pakistan’, ‘Bangladesh’, ‘Sri Lanka’, ‘Myanmar’, ‘Nepal’, ‘Bhutan’, and ‘Afghanistan’. The following data were extracted from the retrieved studies: basic bibliographic details, number of patients who received AHSCT, age of patients, time interval between diagnosis of MM and BMSCT, disease stage, pretransplant disease status, CD34 stem cell yield, engraftment characteristics, TRM, and long-term outcomes (in form of OS and PFS).

## Results

Over a 7.5-year period, 47 patients with MM underwent ASCT (Table 1). The median time to transplant (interval between diagnosis of MM and day 0 of AHSCT) was 11.5 months. G-CSF alone was utilized for peripheral stem cell mobilization in 51% (n=24) and combination therapy with G-CSF and plerixafor was used in the rest. A total of 7.53±2.93×10^8^ mononuclear cells/kg and 5.70 (2.92, 10.43)×10^6^ CD34 cells/kg were harvested. Expectedly, the number of mononuclear cells harvested was higher with G-CSF plus plerixafor as compared to G-CSF alone (median: 8.69×10^8^ vs. 6.42×10^8^ cells/kg, p=0.006). Similarly, the amount of CD34 cells harvested was higher with G-CSF plus plerixafor as compared to G-CSF alone (median: 9.01×10^8^ vs. 4.16×10^8^ cells/kg, p=0.00009). Melphalan was used for conditioning in all the patients. Neutrophil and platelet nadir occurred on day 5 (4, 6) and day 5 (4, 7), respectively. Neutrophil and platelet engraftment occurred on day 10 (9, 12) and day 13 (11, 15), respectively. Successful engraftment was noted in all patients (no engraftment failure); however, engraftment syndrome occurred in 2% (n=1). TRM (day 100) was noted in 4% (n=2). During the cytopenic period, patients were managed with supportive therapy (e.g. repeated SDAP infusions - median no. of units: 3 (2, 4), G-CSF therapy - no. of days: 12 [10, 13.0]). Gastrointestinal toxicity (mucositis) was seen in most of the patients with grade III toxicity being the commonest (40%, n=19). Infections complicated the hospital course in 23% of patients (n=11). Hemodynamic instability occurred in 32% of patients (n=15).

**Table 1:** Disease and treatment characteristics of patients included in our study. ^#^Continuous variables have been expressed as median (25th, 75th percentile) or median (minimum–maximum). Categorical data are expressed as percentages {number of cases}. ^@^Renal impairment was defined as estimated creatinine clearance <60 ml/min/1.73m^2^. BD: bortezomib, dexamethasone; CBD: cyclophosphamide, bortezomib, dexamethasone; CR: complete response; ISS: International Staging System; LBD: lenalidomide, bortezomib, dexamethasone; NA: not applicable; PR: partial response; SD: stable disease; VGPR: very good partial response

Parameter	Value^#^
Age (years)	52.0 (46, 56)
Females	36 {17}
Immunoglobulin subtype	IgG κ: 60 {28}
	IgG λ: 2 {1}
	IgA κ: 9 {4}
	IgA λ: 4 {2}
	Light chain κ: 17 {8}
	Light chain λ: 9 {4}
Stage (according to ISS)	I: 6 {3}
	II: 9 {4}
	III: 85 {40}
Renal impairment^@^	26 {12}
Induction regimen	CBD: 64 {30}
	LBD: 30 {14}
	BD: 6 {3}
Number of cycles	10 (6–13)
Pretransplant response	CR: 21 {10}
	VGPR: 49 {23}
	PR: 28 {13}
	SD: 2 {1}
Time to transplant	11.5 (7–16)
CD34 count (×10^6^ cells/kg)	5.70 (0.37–17.80)
Conditioning regimen	Melphalan: 100 {47}
Post-transplant consolidation	9 {4}
Post-transplant maintenance	Bortezomib: 60 {28}
	Lenalidomide: 32 {15}
	NA: 9 {4}
Duration of maintenance (years)	Bortezomib: 2.0 (0.3–2.6)
	Lenalidomide: 2.5 (0.2–4.4)

**Figure 1:**
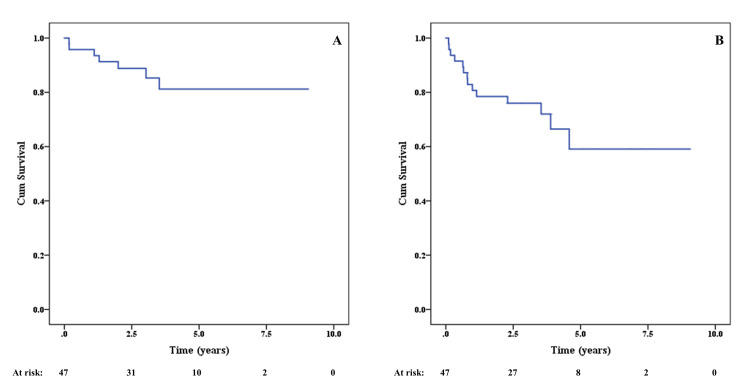
Kaplan-Meier curves depicting the overall survival (panel A) and progression-free survival (panel B) in our study.

The follow-up duration in our study was 3.49±1.99 years with a total follow-up of 164.25 patient-years. A fatal outcome was noted in 15% of patients (n=7). Reasons for death included disease progression (six patients relapsed) and TRM (two patients {sepsis and disease progression, respectively}). The two-year and five-year OS were 88.8% (95% CI 79.6% to 98.0%) and 81.2% (95% CI 68.1% to 94.3%), respectively (Figure 1).

The five-year OS in patients with (pretransplant) complete response/very good partial response (CR/VGPR) and partial response/stable disease (PR/SD) was 85.2% and 72.4%, respectively (p=0.5, Log Rank test). The five-year OS stratified according to the amount of CD34 cells harvested was as follows: <2.0×10^6^ cells/kg: 55.6% and >2.0×10^6^ cells/kg: 85.8%. However, this conspicuous difference in OS did not assume statistical significance (p=0.3, Log Rank test) which was likely due to the small sample size of our study. Other parameters (e.g. sex, MM subtype, disease stage, type of mobilization regimen, presence of kidney disease, etc.) also did not correlate significantly with OS on univariate analysis.

Disease relapse was noted in 28% (n=13). Nearly half of the patients with disease relapse (46%, n=6/13) had succumbed to the illness at the time of last follow-up. PFS at two years and five years was 78.5% (95% CI 66.7% to 90.1%) and 59.1% (95% CI 38.9% to 79.3%), respectively (Figure 1). The five-year PFS was similar in patients with (pretransplant) CR/VGPR (58.9%) compared to PR/SD (62.5%) (p=0.4, Log Rank test). Similarly, patients with stage I/II disease had comparable PFS to patients with stage III disease. The five-year PFS stratified according to the amount of CD34 cells harvested was as follows: <2.0×10^6^ cells/kg: 34.3% and >2.0×10^6^ cells/kg: 63.5%. However, this obvious difference also failed to assume statistical significance (p=0.4, Log Rank test) which was similar to the scenario observed for OS. Other parameters (e.g. sex, MM subtype, type of mobilization regimen, presence of kidney disease, etc.) also did not correlate significantly with PFS on univariate analysis.

Literature review

Literature review of 31 studies from the Indian subcontinent reported utilization of AHSCT in >1700 patients of MM (median age ranging from 46-69 years). Of these, the proportion of patients reported to have renal involvement has been 10-35%. The median time to transplant has ranged from seven to 17 months with median CD34 counts of 2.65-6.3×10^6^ cells/kg. The five-year PFS and OS in these studies have varied from 19.1-75.3% and 50-84.2%, respectively. In studies reporting median survival, PFS and OS have ranged from 2.2-4.8 years and 3.3-10.4 years, respectively. The patient characteristics and transplant outcomes of the individual studies are detailed in Table 2.

**Table 2:** Review of studies on AHSCT in MM from the Indian subcontinent. Note: Stem cell cryopreservation (-80°C) and preinfusion stem cell viability testing were not performed in most of the abovementioned studies ^1 ^Median age of patients at the time of AHSCT. Age range is provided (in square brackets ‘[]’) where mean/median values are unavailable ^2 ^In studies where mean is provided (instead of median), values are marked with an asterisk ‘*’. ^3 ^Disease stage according to the International Staging System (ISS) expressed as a percentage. Studies where Durie-Salmon staging is provided are marked with (#) ^4 ^Values expressed as percentage ^5 ^Time measured in months and expressed as median [range]. ^6 ^Values of CD34 count correspond to 106 cells/kg and are expressed as median [range]. ^7 ^Values expressed as median [range] ^8 ^Values expressed as percentage probability of survival (time frame in years) ^9 ^Total number of patients with engraftment failure ^A ^Manuscript pertains to outcomes of AHSCT for different cancer types. Specific data for patients with MM included in the table ^B1 ^Data regarding characteristics and outcomes of AHSCT in MM from the same center in New Delhi, India, published sequentially at different time points (a total of 7 publications) ^B2 ^Data pertains to the same center from Mumbai, India, at different time points (1 publication and 2 conference abstracts) ^B3 ^Data pertains to the same center from Dhaka, Bangladesh, at different time points (a total of 2 publications) ^C ^Data obtained from published conference abstracts ^D ^Study includes data about both transplanted and untransplanted patients with MM. Specific data regarding patients with MM who underwent AHSCT are included in the table MM: multiple myeloma; AHSCT: autologous hematopoietic stem cell transplantation; PFS: progression free survival; CR: complete response; PR: partial response; SD: stable disease; VGPR: very good partial response; NA: not applicable; TRM: transplant-related mortality; EF: engraftment failure

Authors, Year, Country [Reference]	No. of patients	Age^1, 2^	Disease stage^3^ (Renal involvement^3^)	Pretransplant response^4^	Time to transplant^2, 5^	CD34 count^2, 6^	Days to neutrophil, platelet engraftment^2, 7^	{TRM-30}, [TRM-100]	OS^8^	PFS^8^	EF^9^
Gupta S, et al. 2000, India [61] ^A^^, B1^	17	50	^(#)^IA: 6	CR: 6	NA	NA	NA	NA	~50% (5)	NA	NA
			IIA: 6	PR: 71							
			IIIA: 59	SD+PD: 18							
			(29%)								
Kumar et al., 2003, India [62] ^B1^	50	52	^(#)^IA: 4	CR+VGPR+PR: 60	17.5* [3–129]	5.1* [0.48–12.74] (n=21)	12 [9–24]	[16%]	62% (2.5)	42% (2.5)	7
			IIA: 2								
			IIB: 2								
			IIIA: 62				13 [8–36]				
			IIIB: 30	SD+PD: 40							
			(32%)								
Bagal et al. 2012, India [63] ^B2, C^	61	46	NA	CR: 36	NA	NA	12	8.0%	73% (5)	33% (5)	NA
				VGPR: 5			17				
				PR: 28							
Dolai et al., 2013, India [64] ^C^	11	50.2	II: 45	CR: 45	NA	4.21 [1.6–6.99]	11 [9–17]	NA	NA	NA	NA
			III: 55	VGPR: 45			14 [10–22]				
				PR: 9							
Kayal et al., 2014, India [65] ^B1^	92	51	I: 27.1	CR: 9.7	12.2 [4.3–99.9]	2.9 [0.9–7.67]	10 [8–27]	{3.2%}	50% (5.1)	50% (3)	NA
			II: 28.2	VGPR: 25							
			III: 18.5	PR: 46.7			14 [9–38]	[3.2%]	51.5% (5)	35.5% (5)	
			(18.5%)	SD: 12							
				PD: 6.5							
Kumar et al., 2014, India [66] ^B1^	191	53	I: 37.2	CR: 57.1	10 [2–128]	NA	NA	NA	50% (10.4)	50% (2.8)	NA
			II: 37.7	VGPR: 17.0							
			III: 23.6	PR: 13.1							
			(23.0%)	SD: 4.2							
Ali et al., 2015, Pakistan [67] ^A^	15	NA	NA	NA	NA	NA	NA	{6.7%}	~65% (6)	NA	1
Kumar et al., 2016, India [68] ^B1^	225	53	I: 36.7	CR: 28.0	10 [2–128]	NA	NA	{7.2%}	50% (7.1)	50% (2.7)	NA
			II: 39.4	VGPR: 16.0							
			III: 23.9	PR: 36.4				[8.4%]			
			(20.4%)	SD: 10.2							
				PD: 9.3							
Akter et al., 2017, Bangladesh [69] ^A, B3^	11	49.54*	NA	NA	NA	NA	10	[0%]	NA	NA	NA
							11		5 patients developed relapse and died (all after +400 days)		
Damodar et al., 2017, India [70] ^C^	26	56.6*	I: 11.5	CR: 58	NA	[2.34–10.35]	NA	NA	NA	NA	NA
			II: 31	VGPR: 31							
			III: 58	PR: 11.5							
Gokarn et al., 2017, India [71] ^B2, C^	85	49	I: 31	CR: 33	10.5	NA	NA	NA	91% (3)	58% (3)	NA
			II: 24	VGPR: 39							
				PR: 21							
			III: 43	SD: 1.2							
				PD: 5.9							
Gyi et al., 2017, Myanmar [72] ^A^	6	[53–62]	III: 100	CR+VGPR: 100%	[0.5–5]	[1.45–4.75]	[10–11]	[~0%]	100% (1)	83.3% (1)	NA
			(17%)				[14–18]				
Abeysinghe et al., 2018, Sri Lanka [73] ^A^	17	NA	NA	NA	NA	NA	NA	[0%]	100% (1)	~65% (1)	NA
Aggarwal et al., 2018, India [38]	141	55	I: 24	CR+VGPR: 51.7	7 [3–79]	3.81 [1.41–9.34]	10 [8–27]	[2.1%]	72% (5)	36% (5)	NA
			II: 22								
			III: 22	PR: 48.2			11 [6–56]				
			(10%)								
Khattry et al., 2018, India [74] ^A, B2^	~73	NA	NA	NA	NA	NA	NA	NA	~75% (5)	~50% (5)	NA
Jena et al., 2018, India [75]	5	69	I: 20	CR: 100	NA	6.3 [4.73–13.57]	12 [12–14]	[0%]	NA	NA	NA
			II: 20								
			III: 60				15 [15–18]				
			(20%)								
Malhotra et al., 2018, India [37]	94	53	I: 27	CR: 42	9 [3–33]	5.1 [1.28–16.23]	11.09* [9–18]	{3.2%}	76.7% (6.5)	55.8% (6.5)	1
			II: 22	VGPR: 39							
			III: 51	PR: 5			12.69* [7–39]				
			(25%)	PD: 14							
Naithani et al., 2018, India [76]	50	56	NA	CR: 62	NA	2.65 [1.52–17]	11 [9–14]	{0%}	NA	NA	NA
				VGPR: 6							
			(30%)	PR: 30			11 [9–32]	[2%]			
				SD: 2							
Uday et al., 2018, India [77] ^C^	172	52	I: 21	NA	NA	NA	NA	3.4%	72% (5)	49% (5)	NA
			II: 41								
			III: 48								
			(22%)								
Akter et al., 2019, Bangladesh [78] ^A, B3, C^	4	NA	NA	NA	NA	NA	NA	[0%]	100% (2)	100% (2)	NA
Bafna et al., 2019, India [79] ^C^	50	55	I: 24	CR: 68	NA	NA	NA	4%	50% (3.3)	50% (2.2)	NA
			II: 50	VGPR: 28							
			III: 26	PR: 4							
			(12%)								
Kulkarni et al., 2019, India [80]	245	51	I: 30.6	CR: 19.25	10.5 [3.9–113.4]	4.57 [1.15–23.7]	12 [9–22]	[2.9%]	61.6% (5)	37.2% (5)	1
			II: 35.1	VGPR: 36.82							
			III: 34.3	PR: 37.24			17 [10–44]				
			(~10%)	SD: 4.18							
				PD: 2.51							
Kumar et al., 2019, India [81] ^B1^	349	52	I: 30	CR: 34.1	10 [2–128]	<4: 68.7%, >4: 19.5%	NA	[5.2%]	50% (7.5)	50% (3.4)	5
			II: 35.3	VGPR: 17.2							
			III: 34.7	PR: 32.1					40.4% (10)	28.2% (10)	
			(24.4%)	SD: 7.4							
				PD: 9.2							
Kumar et al. 2019, India [82] ^C^	66	57	I: NA	CR: 41	NA (median no. of cycles 6 [5–7])	NA	11 [9–13]	{~0%}	82.6% (5)	19.1% (5)	NA
			II: NA	VGPR: 48							
			III: 58	PR: 11			11[8–50]				
			(35%)								
Nair et al., 2019, India [83] ^D^	23	52	I: 20	CR: 57	NA	NA	NA	0%	92% (3)	80% (3)	NA
			II: 29	VGPR: 26							
			III: 18	PR: 17							
Kumar et al., 2022, India [54] ^B1^	363	52	I: 28.3	CR+VGPR+PR: 91.2	11.5 [4–67.5]	2.72 [0.3–12.6]	10 [8–37]	[3.6%]	50% (8.3)	50% (4.8)	NA
			II: 35.6								
			III: 36.1	SD+PD: 8.8			12		68.1% (5)	49.0% (5)	
			(21.8%)								
Poudyal et al., 2022, Nepal [84] ^A^	23	NA	NA	NA	NA	>2	NA	[0%]	100% (1)	NA	NA
									3 patients died (at 1.2, 2.3, and 4.4 years)		
Revannasiddaiah et al. 2022, India [85] ^D^	25	NA	NA	NA	NA	NA	NA	NA	~80% (4)	NA	NA
Saeed et al., 2022, Pakistan [86] ^D^	43	NA	NA	NA	NA	NA	NA	NA	NA	50% (4.2)	NA
Sharma et al., 2022, India [87] ^A^	185	NA	NA	NA	NA	NA	NA	NA	82.3% (5)	29.3% (5)	NA
Sood et al., 2022, India [88]	52	55.5	NA	CR+VGPR: 62	NA	6.06 [1.73–31.75]	11	[0%]	84.2% (5)	75.3% (5)	NA
				PR: 38			11.5				
Banday et al., 2023, India [current study]	47	52	I: 6	CR:21	11.5 [7–16]	5.70 [0.37–17.80]	10 [8–20]	[4%]	81.2% (5)	59.1% (5)	0
			II: 9	VGPR: 49							
			III: 85	PR: 28			13 [7–60]				
			(26%)	SD: 2							

## Discussion

AHSCT has emerged as the standard treatment of MM and is increasingly being performed in developing regions of the world. Herein, we report our experience regarding the long-term outcomes of AHSCT in MM and highlight the challenges faced while performing BMSCT. Despite logistic and technical limitations in LMICs, the reported outcomes of AHSCT in MM from various centers, including ours, are encouraging. Further improvement in outcomes of patients with MM in these resource-limited settings is a hopeful future prospect.

Meta-analyses of randomized trials have shown a PFS benefit of early AHSCT in MM [28]. Delaying transplants may result in an enhanced risk of disease relapse that may be more aggressive biologically. Besides, delays may also result in an increased risk of becoming transplant ineligible due to a decrease in performance status and/or the development of additional comorbidities [29]. Also, it has been shown that in high-risk disease AHSCT results in a better OS as there is more chance of attaining a deeper hematological response in the form of negative MRD status [30]. Additionally, AHSCT in general, and particularly in MM, has a very low TRM [31]. In eligible patients with MM in first remission, no treatment modality is as beneficial as AHSCT [32]. Hence, even in the present era of ever-increasing novel therapies including monoclonal antibodies, newer immunomodulatory agents, and CAR T cells, AHSCT is the standard recommendation across all risk groups in newly diagnosed patients with MM [33].

A significant proportion of eligible patients with MM receive AHSCT in developed countries [34]. In a large series from the USA (between the years 2000-2018), ~61% of 2763 eligible patients with MM underwent a transplant in first remission [34]. Data pooled from 97 transplantation centers across Germany (from 1999-2013) reported one-third of patients with MM to have received AHSCT [35]. In stark contrast, <1% of eligible patients with MM undergo AHSCT in developing countries including India [36,37]. At our center, more than 1000 patients were diagnosed with MM from 2010-2018. However, only 47 underwent AHSCT in first remission. Numerous challenges hinder the undertaking of AHSCT in developing countries. Notably, these include severe financial constraints, lack of education, social taboos, and disproportionate procedural fear. Although non-financial constraints can be mitigated to some extent with psychosocial support and counseling, financial barriers are the most difficult to overcome [38].

The primary cause of financial constraints is the lack of universal health coverage policy including the non-availability of health insurance in various LMICs. As such, the expenses are primarily borne by the patients and their immediate family members. With a majority of the population having sub-optimal earnings, financial constraints become an insurmountable hurdle. In India, >50% of the population belongs to the low-income group which includes ~30% living below the poverty line (daily earnings of <2 USD/day) [39]. The estimated average expenditure for AHSCT in federally funded hospitals in India is ~5000 USD [40]. At our center, a waiver of hospital charges resulted in the incurrence of a lower average cost of AHSCT (~3700 USD). A significant proportion of this amount was also borne out of financial support from philanthropic organizations. Despite significant financial constraints in LMICs, early AHSCT is a more feasible (e.g. cost-effectiveness, favorable outcome) approach for the attainment of optimal long-term outcomes in patients with MM. Data from India has shown that the quality-adjusted life year (QALY) gained through AHSCT is 0.7 years with a gradational cost of ~4100 USD per QALY gained [17]. In LMICs, performing an AHSCT in relapsed MM is far more challenging than doing AHSCT in first remission in LMICs. Achieving remission in relapsed settings needs escalation of treatment which may include expensive newer agents like carfilzomib and daratumumab. Besides, patients with relapsed disease often garner far less psychosocial and philanthropic support. Additionally, patients in developing countries may even lose faith in the scientific treatment of MM (especially in the context of widespread availability of alternative indigenous systems of medicine).

Even when an AHSCT is planned, overcoming various constraints mentioned previously invariably results in time delays. The current standard of carrying out AHSCT is after three to six cycles of induction therapy corresponding to about four months [41]. However, our median time to transplant was 11 months (after initial diagnosis). Similar time delays have been noted in other transplant centers in developing countries (Table 2). This time delay invariably results in additional exposure to chemotherapeutic agents (immunomodulatory/alkylating agents) which often results in decreased stem cell harvest [42-44]. As per the International Myeloma Working Group recommendations, a CD34+ stem cell count of ~8×10^6^/kg should be administered with a minimum target of 4×10^6^ cells/kg [45]. The CD34 cell count yield in studies from developing countries is lower (Table 2) than in well-established transplant centers in resourceful settings [46,47]. However, data regarding the adverse impact of lower CD34 counts on outcomes are largely lacking [48,49]. A CD34 count of >2×10^6^ per kg is adequate for engraftment and higher counts of 8-10×10^6^ per kg have been noted to reduce the duration of hospital stay (by decreasing the engraftment time) [50]. Nonetheless, irrespective of the absolute values of CD34 cells harvested, AHSCT in MM results in a better outcome (as compared to untransplanted patients) even when done after 12 months of initial therapy [51-54].

In developed countries, patients undergoing AHSCT in first remission have four-year survival rates of >80% with a median OS of more than eight years [55-57]. In the recently published update of the landmark IFM 2009 trial, patients who underwent AHSCT in first remission had a median PFS of 47.3 months and an eight-year OS of 62% [58]. In another recent multicentric study from Europe, the median PFS was higher at 56.7 months [59]. We observed a five-year PFS and OS of 59.1% and 81.2%, respectively. Similar outcomes have been noted in other studies from the Indian subcontinent (Table 2). Taken together, these findings indicate that despite resource limitations in LMICs, the reported outcomes of AHSCT in MM are comparable to that of the developed world. Lastly, besides long-term outcomes, data regarding the proximate safety of AHSCT (e.g. low rates of TRM) in LMICs are encouraging [60].

Limitations

The retrospective nature of the study design and the small number of patients included are significant limitations of our study. Nonetheless, we have performed a comprehensive review of the characteristics/outcomes of patients with MM treated with AHSCT, highlighting that our findings are similar to other larger cohorts from the Indian subcontinent. Molecular testing for risk stratification was not performed in the majority of the included patients. We did not perform allogenic HSCT (including cord blood HSCT), which is usually employed in relapsed/refractory cases [89,90], in any of our patients with MM. 

## Conclusions

AHSCT has emerged as the therapeutic standard of care for patients with MM as part of the post-induction consolidation phase. While significant advancements have been made in the developed world over the past few decades, centers in resource-limited settings face daunting challenges in providing early AHSCT to eligible patients with MM. Nonetheless, increasing numbers of AHSCTs are being performed in developing countries with encouraging outcomes. The establishment of newer well-equipped transplant centers and augmentation of existing (transplant) services is imperative to improve the outcomes of patients with oncological diseases (including MM) in LMICs. Besides, the incorporation of BMSCT into various sponsored healthcare programs is crucial for accoutering much-needed socio-economic support. In addition, facilities for advanced transplant options in MM (e.g. tandem transplant, allogenic HSCT) need to be developed in the existing transplant centers.
